# Paracrine effects of human amniotic epithelial cells protect against chemotherapy-induced ovarian damage

**DOI:** 10.1186/s13287-017-0721-0

**Published:** 2017-11-28

**Authors:** Qiuwan Zhang, Shixia Bu, Junyan Sun, Minhua Xu, Xiaofen Yao, Kunyan He, Dongmei Lai

**Affiliations:** 1 International Peace Maternity and Child Health Hospital, School of Medicine, Shanghai Jiao Tong University, 145, Guang-Yuan Road, Shanghai, 200030 People’s Republic of China; 2 Institute of Embryo-Fetal Original Adult Disease Affiliated to Shanghai Jiao Tong University School of Medicine, Shanghai, 200030 People’s Republic of China; 3 Key Laboratory of Systems Biomedicine (Ministry of Education), Shanghai Center for Systems Biomedicine, Shanghai Jiao Tong University, Shanghai, 200240 People’s Republic of China

**Keywords:** Human amniotic epithelial cells (hAECs), hAEC-conditioned medium (hAEC-CM), Premature ovarian failure/insufficiency (POF/POI), Human granulosa-lutein (hGL) cells, TGF-β, Smad pathway, Angiogenesis, Apoptosis

## Abstract

**Background:**

Human amniotic epithelial cells (hAECs) are attractive candidates for regenerative medical therapy, with the potential to replace deficient cells and improve functional recovery after injury. Previous studies have demonstrated that transplantation of hAECs effectively alleviate chemotherapy-induced ovarian damage via inhibiting granulose cells apoptosis in animal models of premature ovarian failure/insufficiency (POF/POI). However, the underlying molecular mechanism accounting for hAECs-mediated ovarian function recovery is not fully understood.

**Methods:**

To investigate whether hAECs-secreting cytokines act as molecular basis to attenuate chemotherapy-induced ovarian injury, hAECs or hAEC-conditioned medium (hAEC-CM) was injected into the unilateral ovary of POF/POI mouse. Follicle development was evaluated by H&E staining at 1, 2 months after hAECs or hAEC-CM treatment. In addition, we performed a cytokine array containing 507 human cytokines on hAECs-derived serum-free conditioned medium. Finally, we further investigated whether hAECs could affect chemotherapy-induced apoptosis in primary human granulosa-lutein (hGL) cells and the tube formation of human umbilical vein endothelial cells (hUVECs) via a co-culture system in vitro.

**Results:**

We observed the existence of healthy and mature follicles in ovaries treated with hAECs or hAEC-CM, whereas seriously fibrosis and many atretic follicles were found in the contralateral untreated ovaries of the same mouse. To distinguish cytokines involved in the process of hAECs-restored ovarian function, hAEC-CM was analyzed with a human cytokines array. Results revealed that 109 cytokines in hAEC-CM might participate in a variety of biological processes including apoptosis, angiogenesis, cell cycle and immune response. In vitro experiments, hAECs significantly inhibited chemotherapy-induced apoptosis and activated TGF-β/Smad signaling pathway within primary granulosa-lutein cells in paracrine manner. Furthermore, hAEC-CM was shown to promote angiogenesis in the injured ovaries and enhance the tube formation of human umbilical vein endothelial cells (hUVECs) in co-culture system.

**Conclusions:**

These findings demonstrated that paracrine might be a key pathway in the process of hAECs-mediating ovarian function recovery in animal models of premature ovarian failure/insufficiency (POF/POI).

**Electronic supplementary material:**

The online version of this article (doi:10.1186/s13287-017-0721-0) contains supplementary material, which is available to authorized users.

## Background

Chemotherapeutic agents are widely used to treat various malignancies, and unavoidably cause progressive ovarian damage leading to the reduced fertility in young female patients [1]. There is no satisfying protective strategy to avoid chemotherapy-induced premature ovarian failure/insufficiency (POF/POI). Recently, many studies in the field of regenerative medicine showed that stem cells transplantation might provide an important methodology for restoring ovarian function.

Human amniotic epithelial cells (hAECs) derived from fetal membranes represent a viable source of stem cells. These cells have low immunogenicity and avoid the ethical issues of embryonic stem cells [2]. In vitro, hAECs have been shown to be capable of differentiating into functional hepatocyte-like cells [3], neural cells [4] and cardiomyocyte-like cells [5], which exert regenerative function in injured tissues.

There is mounting evidence that stem cells transplantation alter the local microenvironment in injured tissue by secreting a broad range of trophic and immunomodulatory factors that can be harvested in serum-free conditioned medium (CM), which can rejuvenate or repair the injured cells and tissues [6, 7]. Studies have demonstrated that human amniotic membrane is an inexpensive biological source of matrix, they could secrete multiple cytokines such as epithelial growth factor (EGF), keratinocyte growth factor (KGF), hepatocyte growth factor (HGF), basic fibroblastic growth factor (bFGF), transforming growth factor beta (TGF-β), bone morphogenetic growth protein-4 (BMP-4) and so on [8]. Thus, some transplanted cells could attenuate myocardial infarction depending on their powerful secretory capacity [9]. Additionally, hAEC-CM induced the differentiation of human umbilical cord blood mesenchymal stem cells into dopaminergic neuron-like cells [10] and promoted corneal wound healing in rabbits [11]. In previous studies, we found that hAECs transplantation increased the fertility of POF/POI mice and restored ovarian function by inhibiting granulose cells apoptosis and alleviating inflammatory reaction in chemo-injured ovaries [12]. However, the study showed that only a fraction of grafted hAECs labeled with green fluorescent protein (GFP) migrated into the injured ovary and differentiated into follicle-stimulating hormone receptor (FSHR)-positive granulosa cells [13]. In further study, we found that intraperitoneal injection of hAEC-CM could restore injured ovarian function [14]. Thus, we hypothesize that hAEC-mediated the recovery of ovarian function mainly comes from their paracrine pathway.

In the present study, hAECs or hAEC-CM was injected into ovaries of mice exposed to chemotherapeutic drugs. The paracrine effects of hAECs on the damaged ovaries were examined histologically. Furthermore, the specific components of hAEC-CM were detected by a human cytokines array. Finally, we further investigate whether hAEC-secreting cytokines affect chemotherapy induced-human granulose cells apoptosis and the tube formation of human umbilical vein endothelial cells (hUVECs) in co-culture system.

## Methods

### Isolation and culture of human amniotic epithelial cells (hAECs)

hAECs were isolated as described previously [13]. Human placental tissue was obtained following informed consent from healthy women who tested negative for HIV-I, hepatitis B and hepatitis C. The acquisition protocol was approved by the Institutional Ethics Committee of the International Peace Maternity and Child Health Hospital (IPMCH). Amniotic membranes were mechanically separated from the chorion and dissected into several segments, following phosphate-buffered saline (PBS) washing. The membrane segments were incubated for 25 min at 37 °C with 0.25% trypsin/EDTA (Thermo Fisher Scientific, Waltham, MA, USA). The digested tissue was sequentially filtered through 40 μm filter and centrifuged at 300 g for 5 min at room temperature. Then cells were seeded in 100 mm cell culture plates containing DMEM/F12 (Gibco, Grand Island, NY, USA) supplemented with 10% fetal bovine serum (FBS, Gibco), 2 mM glutamine, streptomycin (100 μg/mL; Gibco), and penicillin (100 U/mL; Gibco). Incubators were set at 37 °C and contained 5% CO_2_.

### Isolation and culture of primary human granulosa-lutein (hGL) cells

The primary human granulosa-lutein cells were obtained from fertility clinic patients following a previously described method [15]. Informed consent was obtained for all participants before their inclusion in this study. This study was approved by the Institutional Ethics Committee of the International Peace Maternity and Child Health Hospital, and written informed consent was obtained from all participants. Follicular fluid (approximately 4–5 ml) was obtained from 20 regularly menstruating healthy women undergoing in vitro fertilization (IVF) due to tubal factor infertility in the IVF Center affiliated to IPMCH Hospital (Shanghai, China). After collection, red blood cells were removed from the follicular fluid by centrifugation at 200 g for 10 min. The dispersed cells were overlaid onto a 50% Percoll solution (Yeasen, Shanghai, China) and centrifuged at 400 g for 20 min. hGL cells precipitated at the Percoll-PBS interface, which were aspirated and resuspended in PBS. hGL cells were cultured in medium DMEM/F12 with 10% FBS. Incubators were set at 37 °C and contained 5% CO_2_.

### Immunofluorescence

hAECs and hGL cells were characterized by using the immunofluorescent labeling technique. Cells were fixed with 4% paraformaldehyde (PFA), and then incubated with the following primary antibodies at 4 °C overnight: EpCam (1:200 dilution; Boster Biological Technology, Pleasanton, CA, USA), E-cadherin (1:200 dilution; Boster Biological Technology) and vimentin (1:200 dilution; Boster Biological Technology) were used for the identification of hAECs. FSHR (1:200 dilution; Boster Biological Technology) and N-cadherin (1:200 dilution; Boster Biological Technology) were used for the identification of hGL cells. After that, cells were incubated with secondary antibody conjugated with Alexa Fluor® 488 (1:200, Thermo Fisher Scientific). Cells were counterstained with DAPI (Thermo Fisher Scientific) and examined under the fluorescence microscope (Leica, Wetzlar, Germany).

### Premature ovarian failure and insufficiency (POF/POI) model establishment

Fifty-eight wild-type C57BL/6 female mice aging from 7 to 8 weeks were obtained from Shanghai Experimental Animal Center of Chinese Academy of Sciences. Mice sorted into the chemotherapy (Cy)-ablated group (Cy, n = 48) were given a single intraperitoneal (i.p.) injection of busulfan (Sigma-Aldrich, St. Louis, MO, USA, 30 mg/kg) and cyclophosphamide (CTX, Sigma-Aldrich,120 mg/kg) to induce premature ovarian failure model, as previously described [12]. Mice in the sham-control group were injected with an equivalent volume of PBS (Sham, n = 10). One week after the injection of chemotherapy drugs, 2 × 10^4^ hAECs resuspensed by PBS in a volume of 10 μl were orthotopically injected into one of the ovaries of chemotherapy-induced POF/POI mice using microinjection needles at laparotomy. Animals were sacrificed for substantial experiments at 13th or 17th week (Additional file 1: Figure S1C). All procedures for animals were approved by the Institutional Animal Care and Use Committee of Shanghai and were performed in accordance with the National Research Council Guide for Care and Use of Laboratory Animals. Efforts were made to minimize animal suffering and limit the number of animals used in the study.

### Preparation of hAEC-conditioned medium (hAEC-CM) and cytokine array

To prepare hAEC-CM, we used a method analogous to the described previously [16, 17]. In brief, hAECs (a total of 2 × 10^6^ cells) were added to a 100 mm plate. Twenty-four hours later, the complete culture medium was replaced with 10 ml serum-free DMEM/F12 medium. After another 24 hours, the supernatant was aspirated gently, filtered through a 0.22 μm filter, transferred to ultrafiltration conical tubes (Amicon Ultra-15 with membranes selective for 3 kDa), and centrifuged to concentrate the hAEC-CM. The final concentration was adjusted to 20 times the concentration of the collected hAEC-CM (Additional file 1: Figure S1C). Control medium (DMEM/F12) was generated in the same way except there were no cells in the plate. Cytokine assay (AAH-BLG-1, Ray Biotech, Norcross, GA, USA) was performed as previously described [16]. In addition, concentrated hAEC-CM (from a total of 2 × 10^4^ cells) or DMEM/F12 was injected into the unilateral ovary of chemotherapy-induced POF/POI mice via microinjection needle in a volume of 10 μl. Animals were sacrificed for substantial experiments at 13th or 17th week (Additional file 1: Figure S1C).

### Histologic evaluation

Ovaries were collected at 1 (13th week) or 2 (17th week) month after injection of hAECs or hAEC-CM, respectively. Ovaries were fixed in Bouin’s solution (containing 5% acetic acid, 9% formaldehyde and 0.9% picric acid), paraffin-embedded and serially sectioned at a thickness of 5 μm. Hematoxylin and eosin (H&E) staining was used to evaluate the morphological structure of the ovary, which was observed under light microscopy. Follicle stage was classified according to the accepted definitions described previously [18]. In brief, blind follicle counts were conducted on every five sections of entire ovaries by two independent researchers. The primordial follicle was defined as granulosa cells surrounding a single fusiform oocyte. The primary follicle was surrounded by at least three granulosa cells, resulting in a cubic shape. The secondary follicle appeared surrounded by at least two layers of granulosa cells with no follicular cavity. The mature follicles (antral follicles) contain at least two layers of granulosa cells and demonstrated evidence of follicular cavity.

### RNA extraction and real-time polymerase chain reaction (PCR)

Total RNA was extracted from homogenized tissue using TRIzol (Invitrogen, Carlsbad, CA, USA). A total of 1 μg RNA of ovary was converted to cDNA using Takara kit (Applied Biosystems Foster City, CA, USA/Takara, Shiga, Japan). Genes of interest were amplified in 7900HT fast real-time PCR system (Applied Biosystems) using the SYBR Green Real-time PCR Master Mix (Applied Biosystems, Takara). PCR primers were designed according to cDNA sequences in the NCBI database (Additional file 2: Table S1). Cycling conditions for the PCR machine were as follows: 95 °C 5 s, 60 °C 30s and 72 °C 30s for 40 cycles. Mouse-GAPDH or human-ACTIN was used as an internal control separately. The 2^-ΔΔCT^ method was employed to determine the relative mRNA expression.

### Co-culture assay

Matrigel® pre-coated Boyden chamber with 0.4 μm pore-sized polycarbonate membrane (Corning Costar, Cambridge, MA, USA) were used in co-culture assays. 1 × 10^5^ hGL cells were cultured in the bottom chambers with complete culture medium (DMEM/F12 1:1; 10% FBS). 1 × 10^5^ hAECs were cultured in the upper insert. hGL cells were incubated with 2 mg/ml CTX (Sigma-Aldrich) for 24 hours, and then co-cultured with hAECs. After another 24 hours, cells and conditioned medium in the bottom chambers were individually harvested and used for the further analysis.

### Cell counting Kit-8 (CCK-8) viability assay

Cell viability was assessed using a CCK-8 detection kit (Dojindo Molecular Technologies, Inc., Kumamoto, Japan), according to the manufacturer’s protocol. After cells were treated with different methods as described above, CCK-8 solution was added per well and then incubated at 37 °C for 1 h. Absorbance was measured at 450 nm using a microplate reader (SpectraMax 190; Eppendorf, Hamburg, Germany).

### Flow cytometry

Cells were harvested and stained with primary labeled antibodies as follow according to the technical data sheet: CD90 (BioLegend, San Diego, CA, USA), CD73 (BioLegend) and OCT3/4 (BioLegend) for 30 min at 4 °C. Then cells were washed with pre-cold PBS and analyzed on the flow cytometry at once.

To analyze the effect of hAECs on chemotherapy-induced apoptosis in hGL cells, 3 × 10^6^ cells from different groups were fixed in 4% PFA for 15 min. Cells were washed three times with PBS and stained with Annexin V-FITC at room temperature for 15 min, and propidium iodide (PI) for 5 min. Cell populations were then analyzed using a FC500 flow cytometer (Beckman Coulter Inc., Miami, FL, USA).

### Western blot analysis

For Western blot, protein lysate from hGL cells were separated on 10% SDS-polyacrylamide gel and transferred to a polyvinyl difluoridine membrane (EMD Millipore, Billerica, MA,USA). Membranes were blocked with 10% non-fat milk in Tris-HCl buffer solution containing 0.1% Tween-20 (TBST) and were separately incubated in the following primary antibodies at 4 °C overnight: caspase 3 (1:1000 dilution; Cell Signaling Technology, (CST) Danvers, MA, USA), Actin (1:10000; Abcam, Cambridge, UK), Smad2 (1:1000 dilution; CST), phospho-Smad2(1:1000 dilution; CST), Smad3 (1:1000 dilution; CST) and phospho-Smad3 (1:1000 dilution; CST). After washing with TBST, membranes were incubated with horseradish peroxidase-conjugated goat anti-rabbit IgG (1:1000; Abcam). Visualization of blots was performed using a standard protocol for ECL (Santa Cruz Biotechnology, Dallas. TX, USA). The relative intensity of protein bands was quantified by digital densitometry Image J software (National Institutes of Health, Bethesda, MD, USA). Level of Actin was used as internal standards.

### Immunohistochemical staining

Ovarian tissue sections collected at 1 month after hAECs or hAEC-CM injection were cut at a thickness of 5 μm. After being incubated with 5% bovine serum albumin for 30 min to block the nonspecific antibody binding sites, the samples were then incubated with the primary antibody against CD34 (1:200 dilution; Abcam) overnight at 4 °C. And then, sections were incubated with corresponding biotinylated secondary antibodies (Vector Laboratories, Burlingame, Ca, USA; 1:200) followed by avidin–biotin–peroxidase (1:200), and the immunoreactivity was visualized with 0.05% diaminobenzidine (DAB, Sigma-Aldrich).

### Tube formation assay

The human umbilical vein endothelial cell line (hUVECs) is kindly provided by Dr. JufuTian (International Peace Maternity and Child Health Hospital, School of Medicine, Shanghai Jiao Tong University, Shanghai, China), which is cultured in the DMEM/F12 supplied with 10% FBS. To assess the pro-angiogenic potency of hAEC-secreting cytokines, 5 × 10^4^ hUVECs were cultured in the bottom chamber in Matrigel-coated 24-well plate (Matrigel, BD Biosciences, San Jose, CA, USA). 5 × 10^4^ hAECs were cultured in the upper insert. And then, hUVECs were co-cultured with hAECs for 4 hours to allow the formation of tube-like structures. The images of tube-like structure were acquired under a light microscope, and the length of tube was measured using WimTube Quantitative Tube Formation Image Analysis Software.

### Statistical analysis

The mean and standard error of the mean (SEM) were calculated for experimental variables. Statistical significance was calculated using GraphPad Prism (GraphPad Software Inc, San Diego, CA, USA). Western blot, quantitative PCR and follicle-counting data were analyzed using two-way ANOVA with the least significant difference (LSD) test. Confidence intervals of 95% were deemed statistically significant. Differences between groups were considered significant when *P* < 0.05.

## Results

### Injection of hAECs or hAEC-CM restored ovarian function in chemotherapy-induced POF/POI mice

The hAECs represented a cobblestone-like morphology (Additional file 1: Figure S1A, a). hAECs expressed the high level of epithelial markers CK19 and E-cadherin, low level of mesenchymal marker N-cadherin, and did not express granulosa cell-specific marker FSHR (Additional file 1: Figure S1A, b). Furthermore, hAECs expressed CD90, CD73 and OCT3/4, were positive for epithelial markers EpCam and E-cadherin, and negative for mesenchymal marker vimentin (Additional file 1: Figure S1A, c-n).

**Fig. 1:**
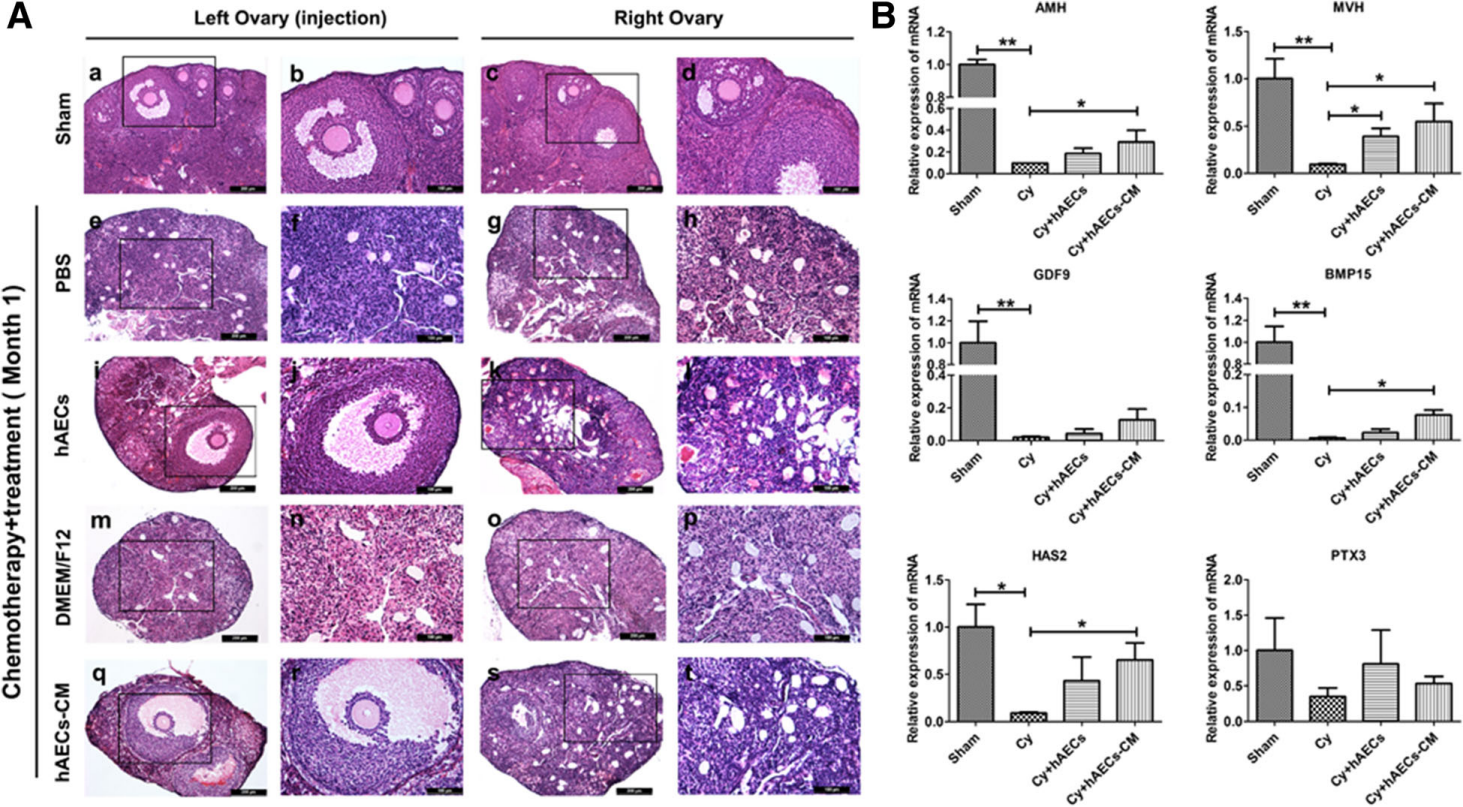
Effects of hAECs and hAEC-CM on follicle development and gene expression in the injured ovaries. **A** Representative photomicrographs of H&E-stained ovarian sections came from left and right ovary obtained from the same mouse in different treatment groups. Pictures b, d, f, h, j, l, n, p, r and t were magnifications of the square in photos a, c, e, g, i, k, m, o, q and s, respectively. **B** Real-time PCR was used to detect the expression of follicle growth-related genes in the injured ovarian tissues at 1 month after hAECs or hAEC-CM treatment, respectively. Data is represented means ± SEM. Sham group, n = 10; Cy + PBS group, n = 10; Cy + hAECs group, n = 10; Cy + DMEM/F12 group, n = 6; Cy + hAEC-CM group, n = 10. ^*^ *P* < 0.05; ^**^ *P* < 0.01. Scale bar is 200 μm in a, c, e, g, i, k, m, o, q and s. Scale bar is 100 μm in b, d, f, h, j, l, n, p, r and t

To further elucidate whether hAEC-mediated the recovery of ovarian function is achieved by improving the local ovarian microenvironment, hAECs or hAEC-CM was injected into the unilateral ovary (left ovary, injection site) using a microinjection needle at 7 days after the administration of chemotherapy drugs (Additional file 1: Figure S1C, a). The morphologic structure of the ovaries was examined by H&E staining. Results showed that many mature and healthy follicles existed in both ovaries of mouse in the sham group (Fig. 1A, a–d). In contrast, many atretic follicles were observed in ovaries from chemo-ablated group (Fig. 1A, e–h and Fig. 1A, m–p). Intriguingly, healthy mature follicles were found in the injured ovaries (left ovary, injection side) at 1 month after hAECs treatment (Fig. 1A, i and j) or hAEC-CM treatment (Fig. 1A, q and r). However, no mature follicles existed in the contralateral ovary (right ovary, un-injection side) of the same mouse (Fig. 1A, k and l and Fig. 1A, s and t).

To further confirm the effects of hAECs or hAEC-CM on ovarian local microenvironment, real-time PCR was performed to compare the expression of genes involved in follicle growth (anti-Müllerian hormone, *AMH*), primordial germ cells (mouse vasa homologue, *MVH*), ovarian folliculogenesis (growth differentiation factor 9, *GDF9*; bone morphogenetic protein 15, *BMP15*) and cumulus expansion (hyaluronic acid synthase 2, *HAS2*; pentraxin 3, *PTX3*) between the treated or untreated ovaries. These results demonstrated that chemotherapy significantly decreased the expression of *AMH, MVH, GDF9, BMP15 and HAS2* in the ovarian tissue. However, hAEC-CM injection significantly increased the expression of *AMH, MVH*, *BMP15* and *HAS2* in chemo-damaged ovaries. hAECs also significantly increased the expression of *MVH* (Fig. 1b). These results indicated that hAECs-secreting cytokines played an important role in hAECs-mediated the recovery of ovarian function after chemotherapy.

### Injection of hAEC-CM or hAECs increased the number of secondary and mature follicles in chemo-injured ovaries

**Fig. 2:**
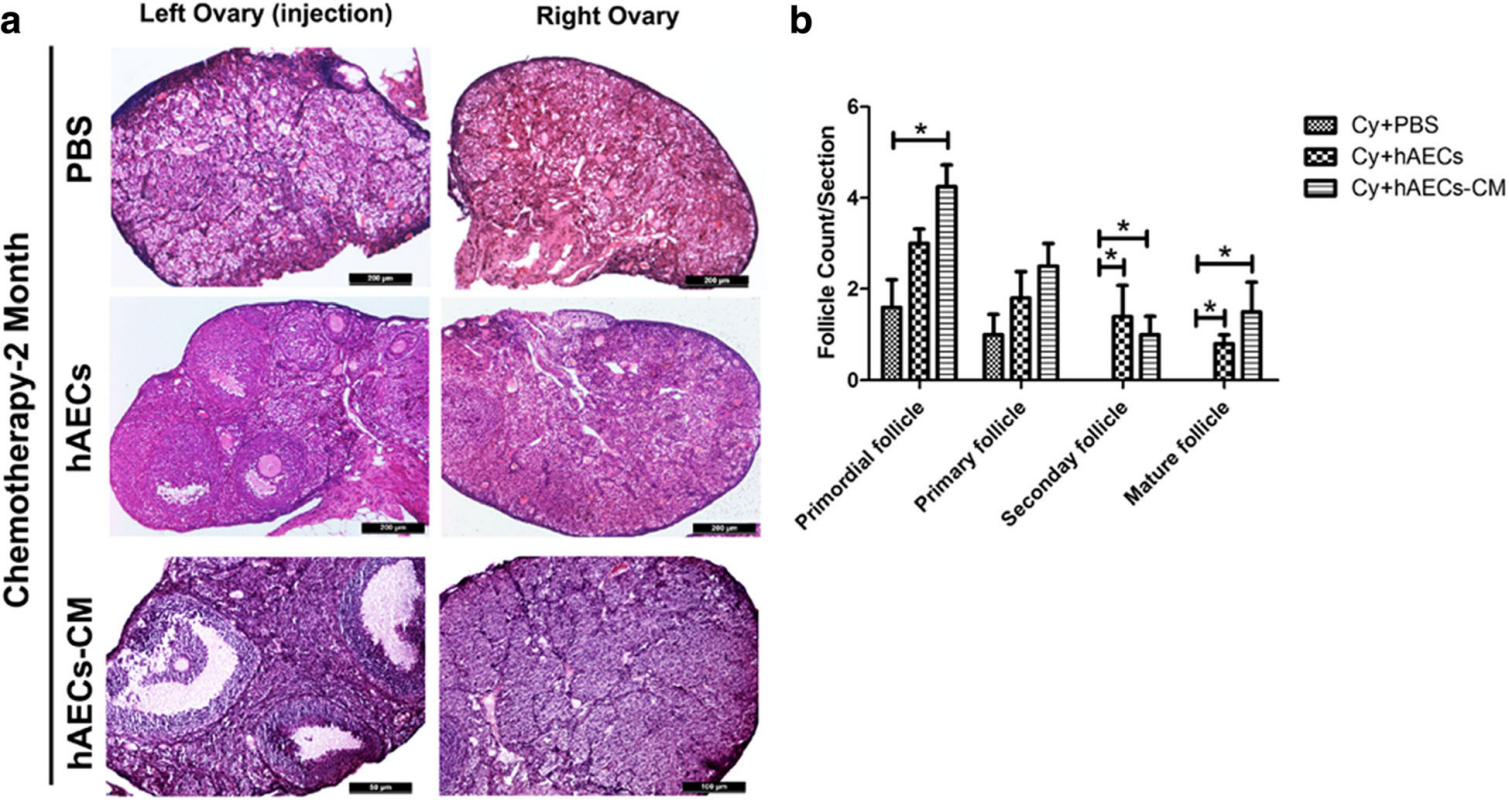
Follicular development and counting in the injured ovaries. **a** Representative images of H&E-stained ovarian sections obtained from different groups at 2 months after hAECs or hAEC-CM treatment. **b** Column displayed the number of primordial, primary, secondary and mature follicles per ovary in different groups. Data represent means ± SEM. Cy group, n = 4; Cy + hAECs group, n = 4; Cy + hAEC-CM group, n = 4. Scale bar is 200 μm in A

In order to investigate the long-term therapeutic potential hAECs and hAEC-CM, we evaluated follicle development at 2 months after hAECs or hAEC-CM treatment, respectively. Histological results showed that many healthy follicles were observed in both hAECs and hAEC-CM injection groups, yet no mature follicles were found in chemotherapy-treated ovaries (Fig. 2a). In addition, the numbers of follicles in different stages were counted in chemo-injured (Cy), chemo-injured/hAEC-treated (Cy + hAECs) and chemo-injured/hAEC-CM treated group (Cy + hAEC-CM). hAECs or hAEC-CM injection increased the number of secondary and mature follicle (*P* < 0.05, Fig. 2b). However, slight effect was observed in the primordial follicles and primary follicles in the chemo-injured ovaries. Only hAEC-CM injection increased the number of primordial follicles (*P* < 0.05, Fig. 2b). Taken together, these results indicated that administration of hAECs or hAEC-CM mainly affected the development of secondary and mature follicles. In addition, hAEC-CM treatment might be effective to promote the development and survival of follicle in injured ovaries.

### Analysis of paracrine components in conditioned medium derived from hAECs

**Fig. 3:**
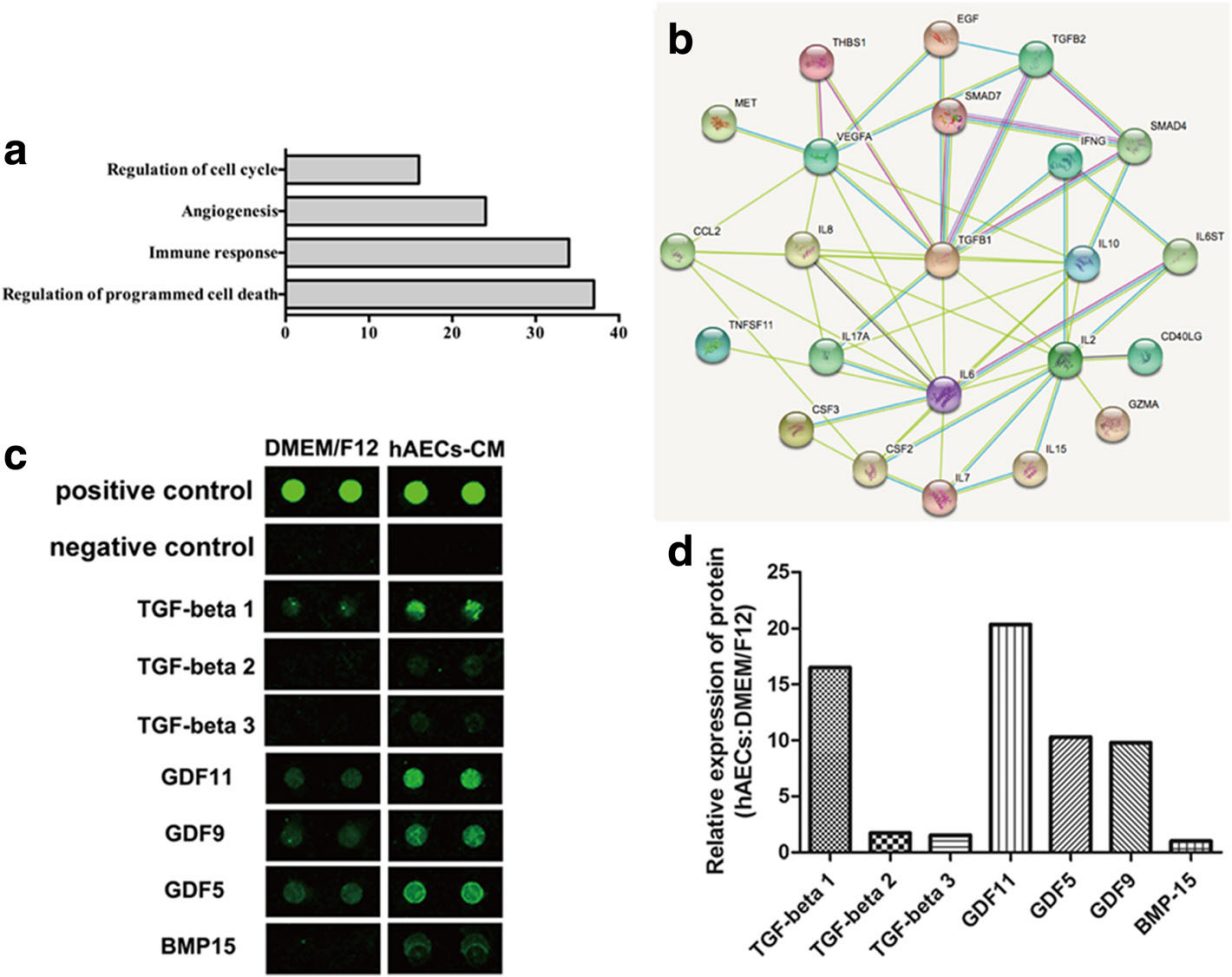
Cytokine array was performed to detect specific cytokines in conditioned medium of hAECs. **a** Gene Ontology (GO) Enrichment Analysis of biological processes, cellular components, and molecular functions demonstrated the number of proteins which might participate in the process of proliferation, immune response, cell apoptosis and angiogenesis. **b** Image of protein and protein interactions and functional network identified by Ingenuity Pathway Analysis (IPA). **c** Selective map of the cytokine array. hAECs released multiple follicular development-associated factors, including TGF-β1/2/3, GDF5/9/11 and BMP15. **d** The intensity of the signals was quantified by densitometry and the expression of BMP15 was regarded as control

To better understand the biological function of hAEC-CM, we detected components in serum-free conditioned medium derived from hAECs using a cytokine array examining 507 human cytokines (DMEM/F12 was used as control). The analytical standard used in this research was set up as follows: (1) The intensity of chemoluminescence intensity should be over 300 in the hAEC-CM group. (2) The chemoluminescence intensity of specific cytokine in the hAEC-CM group should be higher than that in the DMEM/F12 group. (3) The fold change of the ratio of hAEC-CM: DMEM/F12 should be more than two. Then we detected 109 cytokines and ranked them according to the value of hAEC-CM minus DMEM/F12 by chemoluminescence intensity from high to low (Additional file 3: Table S2). According to Gene Ontology Enrichment Analysis (GOEAST), we classified these 109 cytokines into four groups, which are important in the hAECs-mediated the recovery of ovarian function, including regulation of apoptosis (37 proteins), immune response (34 proteins), angiogenesis (24 proteins) and regulation of cell cycle (16 proteins) (Fig. 3a and Additional file 4: Table S3). After analyzing the interactions and functional network identified by the Ingenuity Pathway Analysis (IPA) (using the highest confidence, 0.900), we created a protein-protein interaction network schematic consisting of 23 major cytokines that might contribute to improving ovarian microenvironment in our study (Fig. 3b). Previous studies showed that follicle development was regulated by various factors, such as TGF-β [19], GDF9 and BMP15 [20]. In current study, we found that hAECs secreted multiple follicular development-associated factors, such as GDF5/9/11, TGF-β1/2/3 and BMP15 (Fig. 3c). The relative expression of these cytokines was defined as the ratio of certain cytokine to BMP15, as showed in Fig. 3d.

### hAECs inhibited chemotherapy-induced apoptosis within primary human granulosa-lutein (hGL) cells in co-culture system

**Fig. 4:**
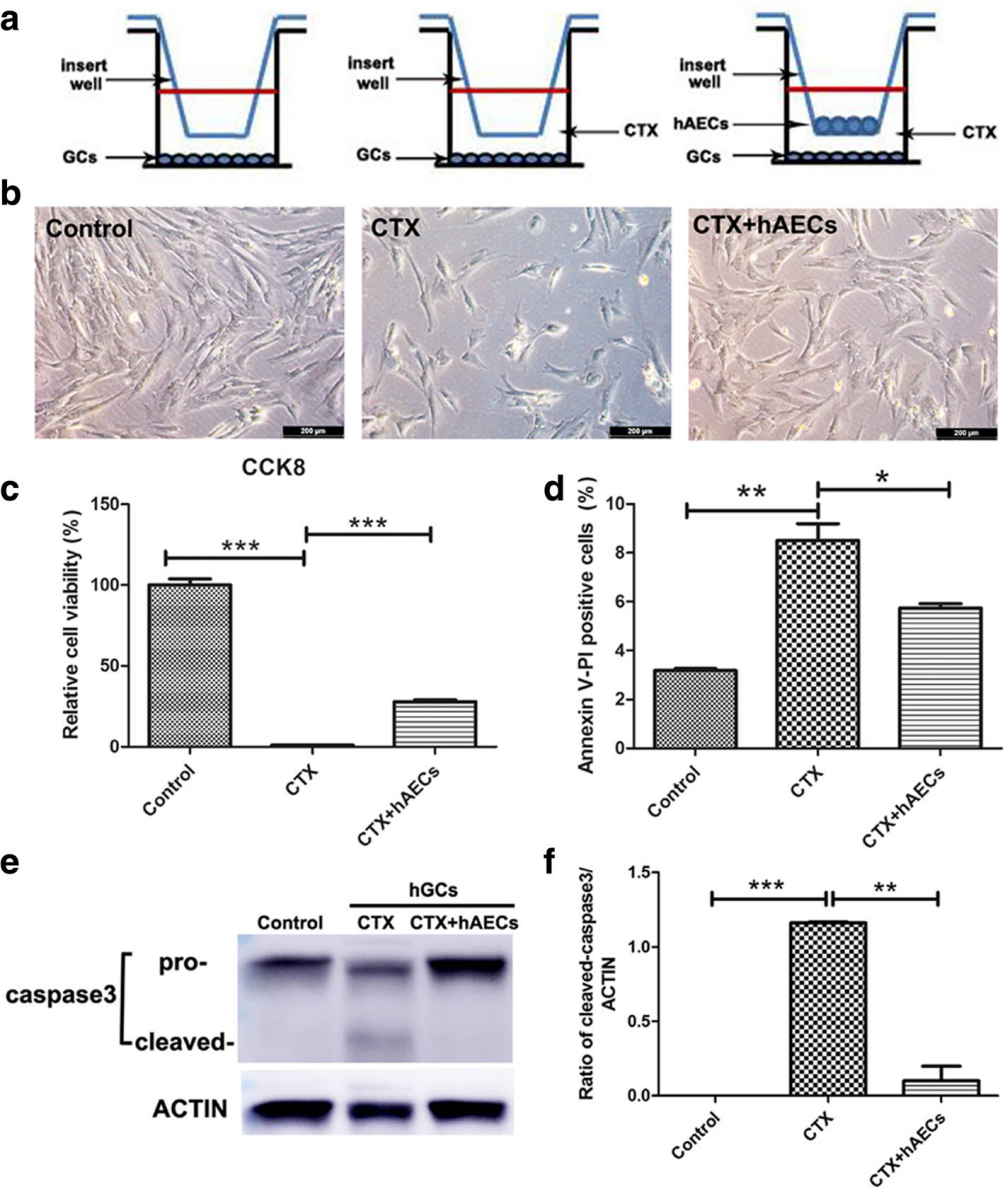
Effect of hAECs-secreting cytokines on CTX-induced apoptosis within human granulosa-lutein (hGL) cells in vitro. **a** 1 × 10^5^ hAECs were seeded on the upper co-culture inserts and 1 × 10^5^ hGL cells were seeded at the bottom of a six-well culture plate with or without CTX. **b** Bright field image of hGL cells on the bottom of co-culture system in different groups. **c** and **d** CCK-8 assay and flow cytometry were used to detect cell viability and apoptosis, respectively. **e** and **f** Western blot was performed to evaluate the level of caspase3 and cleaved caspase3 protein expression in hGL cells. Amount of protein loaded was normalized against actin. Data represent means ± SEM. Control group, n = 4; CTX group, n = 4; CTX + hAECs group, n = 4. ^*^ *P* < 0.05; ^**^ *P* < 0.01; ^***^ *P* < 0.001. Scale bar is 200 μm in B

To investigate the effect of hAEC-CM on chemotherapy-induced granulosa cells damage, primary human granulosa-lutein (hGL) cells were collected from the follicular fluid to perform experiments in vitro. Results displayed that these cells highly expressed granulose cell-specific markers (FSHR and Foxl2) and mesenchymal marker (N-cadherin) as well as the low of epithelial marker (E-cadherin) (Additional file 1: Figure S1B, a-h). And then, we established a co-culture system to elucidate whether hAEC-secreting factors could influence CTX-induced apoptosis in hGL cells (Fig. 4a). Results showed that CTX significantly decreased the viability of hGL cells. However, hAECs could partially reverse chemotherapy drugs-induced cell damage in co-culture systerm (Fig. 4b). CCK-8 cell viability assay also displayed the consistent results with morphology (Fig. 4c). Furthermore, CTX induced dramatically the increased percentage of Annexin-V (+)/PI (+) cells in hGL cells, indicating that these cells were in late-stage apoptosis, whereas this damage could be reversed by co-culture with hAECs (Fig. 4d). In addition, hAECs significantly decreased CTX-induced cleavage of caspase3 expression in paracrine way (Fig. 4e and f). Taken together, primary hGL cells were sensitive to chemotherapy drug. hAECs-secreting cytokines could effectively inhibit chemotherapy-induced apoptosis within granulosa cells in vitro*.*

### hAECs activated the TGF-β/Smad pathway of human luteinized granulosa cells in co-culture system

**Fig. 5:**
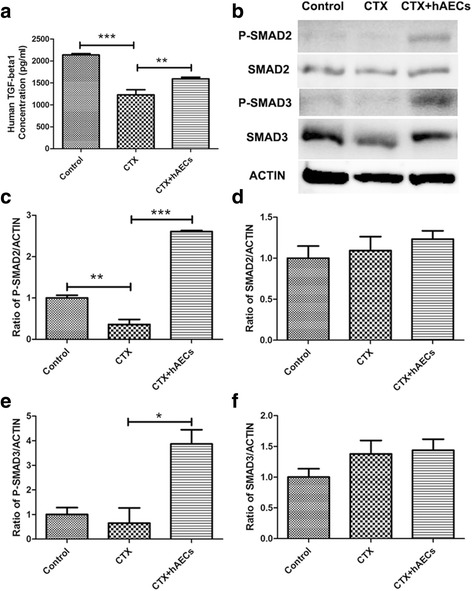
Effect of hAECs-secreting cytokines on TGF-β/SMAD pathway of human luteinized granulosa cells in vitro. **a** ELISA assay was measured TGF-β 1 concentration in conditioned medium derived from GCs, which co-cultured with hAECs for 24 hours. **b**–**f** SMAD protein expressions in human GCs were detected by Western blot after co-cultured with hAECs for 24 hours. hAEC-secreting cytokines activated the expression of P-SMAD2 and P-SMAD3 in granulosa cells. Data represent means ± SEM. Control group (n = 4); CTX group (n = 4); CTX + hAECs group (n = 4). ^*^ *P* < 0.05; ^**^ *P* < 0.01; ^***^ *P* < 0.001

The development of follicles is regulated by various factors, such as TGF-β [19], GDF9 and BMP15 [20]. According to cytokine array results, we further investigate whether hAECs restore ovarian function via activating Smad pathway in granulosa cells. In co-culture system, we detected a high concentration of TGF-β 1 in hGL cells-conditioned medium at 24 hours after co-culture with hAECs comparing with that of CTX-treated group (Fig. 5a). Simultaneously, we collected human luteinized granulosa cells to detect P-SMAD and SMAD proteins expression by Western blot. P-SMAD2 and P-SMAD3 protein expression in granulose cells co-culture with hAECs were significantly higher than CTX-treated group (Fig. 5b–f). These results indicated that hAECs-secreting cytokines could activate Smad pathway of human luteinized granulosa cells in vitro.

### hAECs promoted angiogenesis and vasoformation in the injured ovaries in paracrine manner

**Fig. 6:**
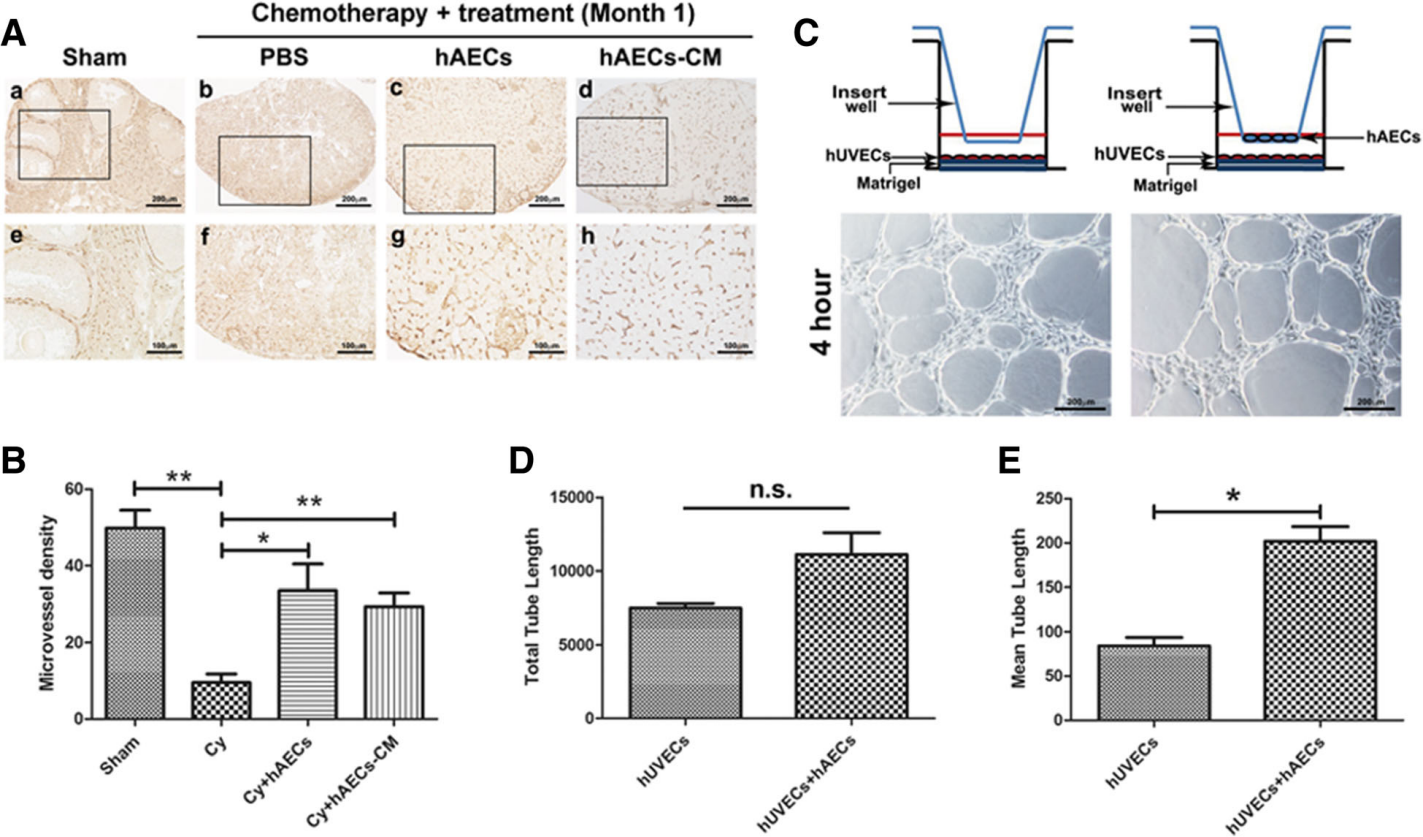
Effect of hAEC-secreting cytokines on angiogenesis and the tube formation of hUVECs in vitro. **A** Histochemical photographs displayed CD34-labeled cells in the injured ovaries of different groups at 1 month after microinjection of hAECs or hAECs-CM. Photographs e, f, g and h were magnifications of the square in photos a, b, c and d, respectively. **B** Quantification showed the density of CD34-positive microvessel in injured ovary of different groups. **C** 5 × 10^4^ hUVECs were seeded on the bottom of a 24-well Matrigel-coated culture plate, and 5 × 10^4^ hAECs were seeded on 0.4 μm pore-size transwell inserts. Bright field image showed the tube-like structure of hUVECs on the bottom of co-culture system at 4 hours after culture with hAECs. **D** and **E** Quantitative analysis demonstrated that paracrine effect of hAECs on tube formation of hUVECs in a co-culture system. Data represent means ± SEM. Sham group (n = 6); Cy group (n = 6); Cy + hAECs group (n = 6); Cy + hAECs-CM group (n = 6); hUVECs group (n = 4); hUVECs and hAECs co-culture group (n = 4). Scale bar is 200 μm in A-a to A-d. Scale bar is 100 μm in A-e to A-h. Scale bar is 200 μm in C

It is well established that chemotherapy irreversibly induced a loss of follicles and decreased microvascularization of the corpora lutea and follicles in a dose-dependent manner [21]. In the current study, we also evaluated the long-term effect of hAECs or hAEC-CM on the number of CD34-positive cells in injured ovaries in vivo and the tube formation of hUVECs in vitro. The microvessel density of CD34-positive cells in the Cy-treated group was significantly lower than that in Sham group. In contrast, hAECs or hAEC-CM significantly increased the density of microvessel at month 1 after treatment (Fig. 6a–b). Although, hAEC-secreted factors did not impact the total tube length of hUVECs statistically, we observed that hAECs significantly increased the mean tube length of hUVECs in co-culture system (Fig. 6c–e).

## Discussion

Fertility preservation is a critical consideration for young female cancer patients after receiving chemotherapy. In this study, we used cyclophosphamide and busulfan to establish the chemotherapy-induced POF/POI mouse model, leading to the activation of quiescent primordial follicle and the reduced of ovarian function [22]. Transplantation of stem cells, including human amniotic epithelial cells [12], human endometrial mesenchymal stem cells [23], skin-derived mesenchymal stem cells [24] and human amniotic fluid stem cells [25], holds great promise as a potential preventive strategy to reduce ovarian injury in female patients accepting chemotherapy. However, the underlying molecular mechanism is still unclear. In a previous study, we found that direct trans-differentiation of hAECs into granulosa cells in the injured ovary was a rare event and might play only a minor role [13], indicating that hAEC-secreting cytokines might play more important role in the hAEC-mediated ovarian function recovery. In current study, we observed that injection of hAEC-CM significantly increased the number of secondary and mature follicles, as well as upregulated follicle growth-related genes expression (*AMH, MVH, BMP15* and *HAS2*). This discrepancy might be that secretary function and viability of hAECs in the condition of normal culture in vitro is different from chemotherapy-induced pathological environment in vivo.

A growing body of evidence suggests that stem cells promote the recovery of injured tissue via secreting factors [26–28]. To investigate the specific molecular mechanism in the process of hAEC-mediated ovarian function repair/regeneration, we examined the potentially functional components in hAEC-CM and mainly focused on some important cytokines, which were predicted to engage in diverse biological processes such as controlling cell cycle progression, regulating immune response, impacting apoptosis and angiogenesis. In the current study, we have confirmed that cytokines secreted by hAECs reduce the expression of cleaved caspase3 protein, and promote angiogenesis against CTX-induced damage in vivo and in vitro*.*

TGF-β superfamily has been proved to be important to ovarian function and microenvironment. TGF-β signaling participates in regulating follicular development [29–31], follicle recruitment [32], ovulation [33] and lutenization [34]. In addition, it is vital to regulate steroidogenesis in granulosa cells [35, 36] and impact the expression of follicular development-related genes, such as connexin43 [37], connective tissue growth factor [34], Cyclooxygenase-2 and prostaglandin E2 [38]. Furthermore, we found that hAEC-secreting factors exerted restorative function on chemotherapy-induced vascular injury in vivo and protective ability against CTX-induced apoptosis in human granulosa cells in vitro.

The mechanism of hAECs-mediated ovarian function recovery after chemotherapy is complicated. Although TGF-β superfamily plays an important role in the process of follicle development and function recovery, the powerful secretive ability of hAECs should be further investigated. Exosomes are small vesicles that originate from endocytic multivesicular compartments and are secreted by a variety of cell types, which mediate cell-to-cell or cell-to-environment communication [39]. It is known that multiple microRNAs are involved in the formation of primordial follicles, follicular recruitment and selection, follicular atresia, oocyte-cumulus cell interaction, granulosal cell function and luteinization [40]. Study has reported that exosomal microRNA10a derived from amniotic fluid stem cells preserves ovarian follicles after chemotherapy [41]. Our further study has confirmed the existence of hAEC-derived exosomes (data not shown). Thus, these specific microRNAs in hAECs-exosomes and their function in the process of ovarian function recovery will be elucidated in the future study.

**Fig. 7:**
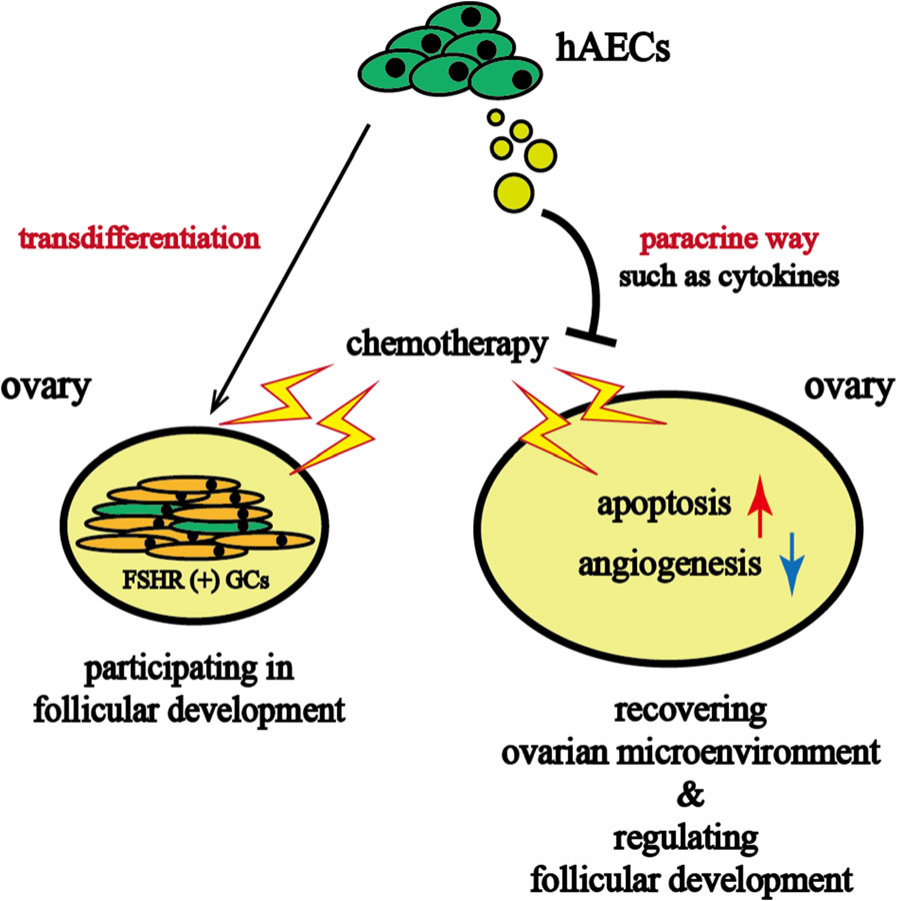
Schematic diagram illustrated the two possible mechanisms responsible for the restoration effects of hAECs-based therapy. (1) *Left side* showed the transdifferentiation ability of hAECs into FSHR-positive granulosa cells in chemotherapy-induced POF/POI model, which was considered as a small probability event. (2) *Right side* showed that hAECs-secreting cytokines exerted protective and restorable function on ovarian microenvironment against chemotherapy-induced damage via reducing apoptosis, promoting angiogenesis and regulating follicular development

The present study demonstrated the presence of paracrine pathway accounting for hAEC-mediating recovery of ovarian function through inhibiting apoptosis and stimulating angiogenesis in the chemotherapy-injured ovaries. Based on our previous studies [12–14], we propose two possible mechanisms for the beneficial effects of hAEC-based strategy in chemotherapy-induced POF/POI model as follows: (1) A small quantity of hAECs differentiate into granulosa cells and participate in follicular development. (2) hAEC-secreting cytokines improve the injured ovarian microenvironment, including inhibiting chemotherapy-induced apoptosis, promoting angiogenesis and regulating follicular development (Fig. 7). This paradigm may offer novel insights into potential mechanism underlying the protective function of hAECs against chemotherapy-induced ovarian damage.

## Conclusions

This study suggests that hAECs may offer a viable method for preventing and/or treating chemotherapy-induced ovarian injury. Moreover, paracrine pathway plays a vital role in hAECs-based recovery of ovarian function depending on the fact that hAEC-CM produced a comparable and potentially better effect. The protective effect of hAEC-CM is associated with some enriched important cytokines, such as TGF-β1, GDF9, BMP15 which involve in the process of anti-apoptosis, regulation of follicle development and pro-angiogenesis in the injured ovary. These novel insights offer a clue to the potential mechanism underlying hAEC-mediating ovarian function recovery, which may be able to preserve the fertility in female cancer patients.

## Additional files

Additional file 1: Figure S1. Characterization of hAECs and hGL cells. (A-a) Morphology of hAECs. (A-b) Real-time PCR showed the expression of epithelial markers (CK19 and E-cadherin), mesenchymal marker (N-cadherin) and granulosa cell-specific marker (FSHR) in hAECs from four clinical samples. (A-c to A-e) Flow cytometry was used to test stem cell markers (CD90, CD73 and OCT3/4) in hAECs. (A- f to A-n) Immunofluorescence displayed the expression of epithelial markers (EpCam and E-cadherin), and mesenchymal marker (vimentin) in hAECs. (B-a) Morphology of hGL cells. (B-b) Real-time PCR was used to test expression of epithelial marker (E-cadherin), mesenchymal marker (N-cadherin) and hGL cell-specific markers (FSHR and Foxl2) in hGL cells from four clinical samples. (B-c to B-h) Immunofluorescence showed the expression of FSHR and mesenchymal marker (N-cadherin) in hGL cells. (C-a) The workflow of animal experiments conducted in this study. C57BL/6 female mice aging from 8 weeks were intraperitoneal injected with chemotherapy (30 mg/kg busulfan and 120 mg/kg cyclophosphamide). PBS, 2 × 10^4^ hAECs or centrifuged hAEC-CM from 2 × 10^4^ hAECs was injected into one of the ovary of chemotherapy-induced POF/POI mice via microinjection needle. Animals were sacrificed for substantial experiments at 13th or 17th week. (C-b) The procedure of production centrifuged condition medium from hAECs. Scale bar is 100 μm in A-f to A-n. Scale bar is 200 μm in B-c to B-h. (TIF 7959 kb)

Additional file 2: Table S1. PCR primers used to detect gene expression in tissue and cells. Mouse (*m*), human amniotic epithelial cells (*h*) and human granulosa-lutein cells (*h*). (DOCX 15 kb)

Additional file 3: Table S2. This list showed the 109 enriched cytokines in conditioned medium of hAECs. (DOCX 29 kb)

Additional file 4: Table S3. This list showed the enriched cytokines in hAECs conditioned medium. These cytokines participate in the regulation of apoptosis (37 cytokines), immune response (34 cytokines), angiogenesis (24 cytokines), or cell cycle progression (16 cytokines). (DOCX 20 kb)

## Abbreviations

AMH: Anti-Müllerian hormone
bFGF: Basic fibroblastic growth factor
BMP-15: Bone morphogenetic growth protein-15
BMP-4: Bone morphogenetic growth protein-4
CCK-8: Cell counting kit-8
CM: conditioned medium
CTX: Cyclophosphamide
Cy: Chemotherapy
EGF: Epithelial growth factor
FSHR: Follicle-stimulating hormone receptor
GCs: Granulosa cells
GDF9: Growth differentiation factor 9
GFP: Green fluorescent protein
H&E: Hematoxylin and eosin
hAEC-CM: Human amniotic epithelial cells-conditioned medium
hAECs: Human amniotic epithelial cells
HAS: Hyaluronic acid synthase
HGF: Hepatocyte growth factor
hGL: Human granulosa-lutein
hUVECs: Human umbilical vein endothelial cells
KGF: Keratinocyte growth factor
MVH: Mouse vasa homologue
PBS: Phosphate-buffered saline
PCR: Polymerase chain reaction
PFA: Paraformaldehyde
POF/POI: Premature ovarian failure/insufficiency
PTX: Pentraxin
TGF-β: Transforming growth factor beta
