# Supplementary figures and images for: Paracrine effects of human amniotic epithelial cells protect against chemotherapy-induced ovarian damage
Source: Stem Cell Res Ther. 2017 Nov 28;8:270. doi: 10.1186/s13287-017-0721-0 (PMC5704397; doi:10.1186/s13287-017-0721-0)

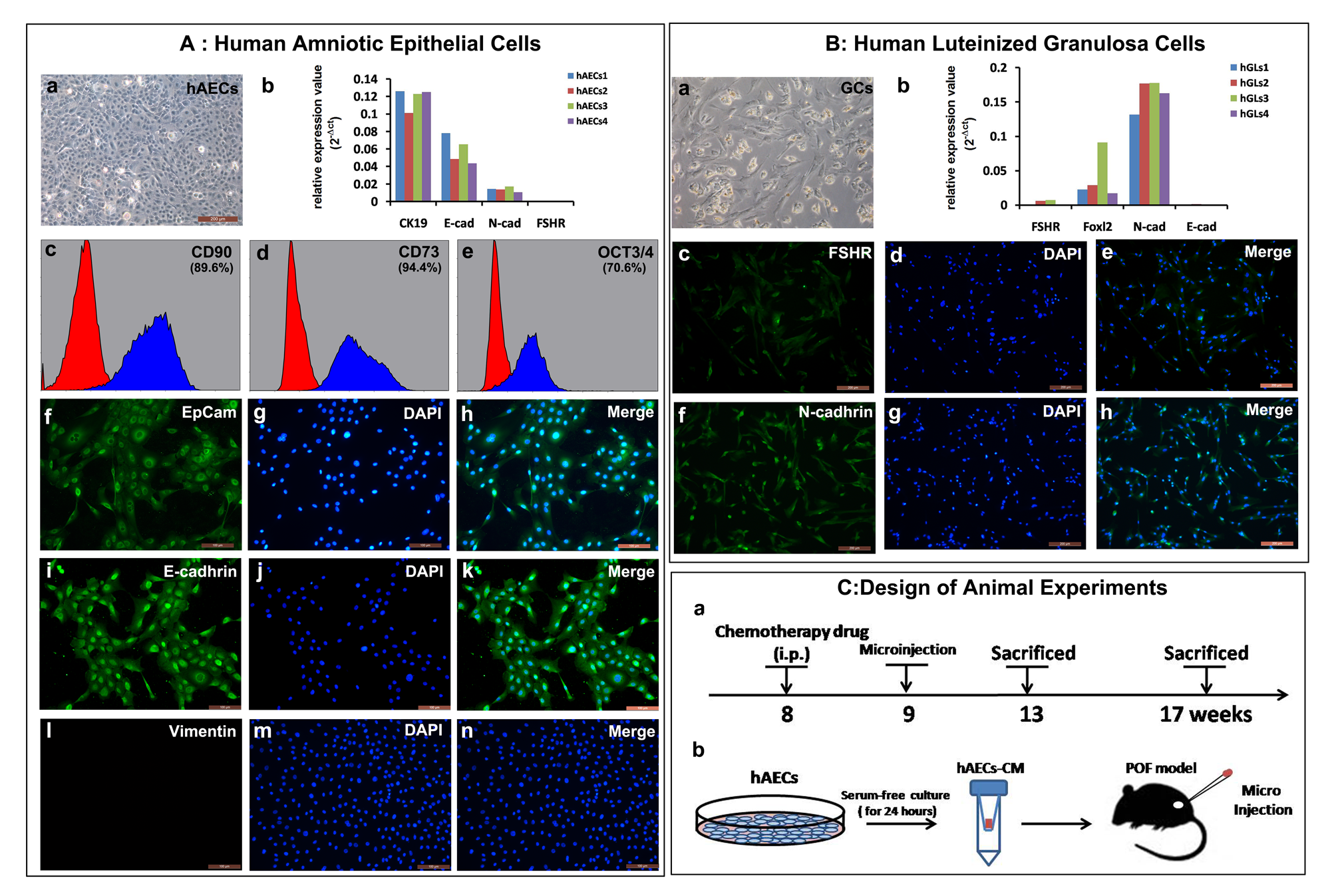

Supplement: Supplementary file 1 — Characterization of hAECs and hGL cells. (A-a) Morphology of hAECs. (A-b) Real-time PCR showed the expression of epithelial markers (CK19 and E-cadherin), mesenchymal marker (N-cadherin) and granulosa cell-specific marker (FSHR) in hAECs from four clinical samples. (A-c to A-e) Flow cytometry was used to test stem cell markers (CD90, CD73 and OCT3/4) in hAECs. (A- f to A-n) Immunofluorescence displayed the expression of epithelial markers (EpCam and E-cadherin), and mesenchymal marker (vimentin) in hAECs. (B-a) Morphology of hGL cells. (B-b) Real-time PCR was used to test expression of epithelial marker (E-cadherin), mesenchymal marker (N-cadherin) and hGL cell-specific markers (FSHR and Foxl2) in hGL cells from four clinical samples. (B-c to B-h) Immunofluorescence showed the expression of FSHR and mesenchymal marker (N-cadherin) in hGL cells. (C-a) The workflow of animal experiments conducted in this study. C57BL/6 female mice aging from 8 weeks were intraperitoneal injected with chemotherapy (30 mg/kg busulfan and 120 mg/kg cyclophosphamide). PBS, 2 × 10^4^ hAECs or centrifuged hAEC-CM from 2 × 10^4^ hAECs was injected into one of the ovary of chemotherapy-induced POF/POI mice via microinjection needle. Animals were sacrificed for substantial experiments at 13th or 17th week. (C-b) The procedure of production centrifuged condition medium from hAECs. Scale bar is 100 μm in A-f to A-n. Scale bar is 200 μm in B-c to B-h. (TIF 7959 kb) [file 13287_2017_721_MOESM1_ESM.tif]
