# Supplementary material for: Paracrine effects of human amniotic epithelial cells protect against chemotherapy-induced ovarian damage
Source: Stem Cell Res Ther. 2017 Nov 28;8:270. doi: 10.1186/s13287-017-0721-0 (PMC5704397; doi:10.1186/s13287-017-0721-0)
Supplement: Supplementary file 2 — PCR primers used to detect gene expression in tissue and cells. Mouse (m), human amniotic epithelial cells (h) and human granulosa-lutein cells (h). (DOCX 15 kb) [file 13287_2017_721_MOESM2_ESM.docx]

Additional file 2: Table S1. PCR primers used to detect gene expression in tissue and cells. Mouse (m), human amniotic epithelial cells (h) and human granulosa-lutein cells (h).

| **Gene symbol** | **Primer sequence** | | | **Fragment**  **size (bp)** |
| --- | --- | --- | --- | --- |
| mAMH | Forward: | GGGGAGACTGGAGAACAGC |  | 67 |
|  | Reverse: | AGAGCTCGGGCTCCCATA |  |  |
| mMVH | Forward: | CAAGCGAGGTGGCTGCCAAG |  | 194 |
|  | Reverse: | CTGAATCACTTGCTGCTGGTTTCC |  |  |
| mGDF9 | Forward: | AGTCACCTCTACAATACCGTCCG |  | 148 |
|  | Reverse: | CCGATTTGAGCAAGTGTTCCA |  |  |
| mBMP15 | Forward: | CAGTAAGGCCTCCCAGAGGT |  | 113 |
|  | Reverse: | AAGTTGATGGCGGTAAACCA |  |  |
| mHAS2 | Forward: | AAGACCCTATGGTTGGAGGTGTT |  | 167 |
|  | Reverse: | CATTCCCAGAGGACCGCTTAT |  |  |
| mPTX3 | Forward: | GGACAACGAAATAGACAATGGACTT |  | 109 |
|  | Reverse: | CGAGTTCTCCAGCATGATGAAC |  |  |
| mGAPDH  hCK19  hE-cad  hN-cad  hFSHR  hFoxl2  hACTIN | Forward: | CCAATGTGTCCGTCGTGGATCT |  | 149  211  131  120  131  60  250 |
|  | Reverse:  Forward:  Reverse:  Forward:  Reverse:  Forward:  Reverse:  Forward:  Reverse:  Forward:  Reverse:  Forward:  Reverse: | GTTGAAGTCGCAGGAGACAACC  TTTGAGACGGAACAGGCTCT  AATCCACCTCCACACTGACC  GCCTCCTGAAAAGAGAGTGGAAG  TGGCAGTGTCTCTCCAAATCCG  TTTGATGGAGGTCTCCTAACACC  ACGTTTAACACGTTGGAAATGTG  TCTGTCACTGCTCTAACAGGG  TGCACCTTTTTGGATGACTCG  TCCAATAAAGATGGTTTCGTCT  TCTTCAGATAGGGAGAGGGTGA  CATGTACGTTGCTATCCAGGC  CTCCTTAATGTCACGCACGAT |  |  |
