# Supplementary material for: Paracrine effects of human amniotic epithelial cells protect against chemotherapy-induced ovarian damage
Source: Stem Cell Res Ther. 2017 Nov 28;8:270. doi: 10.1186/s13287-017-0721-0 (PMC5704397; doi:10.1186/s13287-017-0721-0)
Supplement: Supplementary file 3 — This list showed the 109 enriched cytokines in conditioned medium of hAECs. (DOCX 29 kb) [file 13287_2017_721_MOESM3_ESM.docx]

**Additional file 3: Table S2**. This list showed the 109 enriched cytokines in conditioned medium of hAECs.

| **No.** | **Protein name** | **Gene name** | **Accession number** | **CI of**  **DMEM/12** | **CI of**  **hAECs-CM** | **hAECs-CM /**  **DMEM/F12** | **hAECs-CM -**  **DMEM/F12** |
| --- | --- | --- | --- | --- | --- | --- | --- |
| 1 | Thrombospondin-1 | THBS1 | NM_005930 | 153 | 28,255 | 184.37 | 28,102 |
| 2 | MIF | MIF | NM_002415 | 50 | 8,172 | 164.26 | 8,122 |
| 3 | GCSF | CSF3 | NM_001742 | 1,303 | 7,171 | 5.50 | 5,868 |
| 4 | IL-2 | IL2 | NM_000586 | 1 | 5,857 | 5856.68 | 5,856 |
| 5 | TNF-beta | LTB | NM_002341 | 1,084 | 6,864 | 6.34 | 5,780 |
| 6 | Granzyme A | GZMA | NM_006144 | 1,124 | 6,901 | 6.14 | 5,777 |
| 7 | GDF11 | GDF11 | NM_005811 | 1,067 | 6,815 | 6.39 | 5,748 |
| 8 | MCP-1 | CCL2 | NM_002982 | 953 | 5,937 | 6.23 | 4,984 |
| 9 | IL-21 | IL21 | NM_021803 | 1,166 | 5,999 | 5.15 | 4,833 |
| 10 | GRO | CXCL1 | NM_005222 | 1,081 | 5,782 | 5.35 | 4,701 |
| 11 | TGF-beta 1 | TGFB1 | NM_000660 | 995 | 5,653 | 5.68 | 4,658 |
| 12 | CCL28 / VIC | CCL28 | NM_148672 | 506 | 5,067 | 10.01 | 4,560 |
| 13 | GM-CSF | CSF2 | NM_000758 | 1,065 | 5,417 | 5.09 | 4,352 |
| 14 | FGF-16 | FGF16 | NM_003868 | 453 | 4,710 | 10.40 | 4,257 |
| 15 | IL-15 | IL15 | NM_001996 | 894 | 5,080 | 5.68 | 4,186 |
| 16 | IL-8 | IL8 | NM_021727 | 424 | 4,495 | 10.62 | 4,072 |
| 17 | IFN-gamma | IFNG | NM_000619 | 944 | 4,961 | 5.26 | 4,017 |
| 18 | IL-7 | IL7 | NM_000880 | 337 | 4,281 | 12.72 | 3,944 |
| 19 | IL-13 | IL13 | NM_002188 | 654 | 4,584 | 7.01 | 3,931 |
| 20 | IL-10 | IL10 | NM_000572 | 1,068 | 4,959 | 4.65 | 3,892 |
| 21 | IL-6 | IL6 | NM_000600 | 819 | 4,594 | 5.61 | 3,775 |
| 22 | MIG | CXCL9 | NM_002416 | 832 | 4,390 | 5.28 | 3,558 |
| 23 | Insulin R | INSR | NM_000208 | 1,054 | 4,512 | 4.28 | 3,458 |
| 24 | Orexin B | HCRTR2 | NM_017434 | 240 | 3,149 | 13.15 | 2,910 |
| 25 | GDF5 | GDF5 | NM_000557 | 1,212 | 4,120 | 3.40 | 2,908 |
| 26 | IGFBP-3 | IGFBP3 | NM_000598 | 113 | 2,915 | 25.85 | 2,802 |
| 27 | GDF9 | GDF9 | NM_021080 | 933 | 3,700 | 3.97 | 2,767 |
| 28 | IGFBP-rp1 / IGFBP-7 | IGFBP7 | NM_001553 | 113 | 2,326 | 20.67 | 2,213 |
| 29 | TNF-alpha | LTA | NM_000595 | 753 | 2,719 | 3.61 | 1,966 |
| 30 | HGFR | MET | NM_000245 | 1,204 | 3,161 | 2.63 | 1,957 |
| 31 | IL-1 alpha | IL1A | NM_000575 | 814 | 2,674 | 3.29 | 1,861 |
| 32 | Ubiquitin+1 | UBA52 | NM_003333 | 419 | 1,975 | 4.71 | 1,556 |
| 33 | Latent TGF-beta bp1 | LTBP1 | NM_206943 | 115 | 1,630 | 14.17 | 1,515 |
| 34 | Endothelin1 | EDN1 | NM_001955 | 801 | 2,250 | 2.81 | 1,450 |
| 35 | Endothelin2 | EDN2 | NM_001956 | 801 | 2,250 | 2.81 | 1,450 |
| 36 | Endothelin3 | EDN3 | NM_207034 | 801 | 2,250 | 2.81 | 1,450 |
| 37 | EDG-1 | EDG1 | NM_001400 | 633 | 2,016 | 3.18 | 1,383 |
| 38 | Erythropoietin | EPO | NM_000799 | 715 | 1,935 | 2.71 | 1,220 |
| 39 | TMEFF2 | TMEFF2 | NM_016192 | 66 | 1,255 | 19.01 | 1,189 |
| 40 | NRG3 | NRG3 | NM_000450 | 948 | 2,108 | 2.22 | 1,160 |
| 41 | Progranulin | GRN | NM_001931 | 13 | 1,173 | 93.82 | 1,160 |
| 42 | FGF-17 | FGF17 | NM_003768 | 576 | 1,714 | 2.98 | 1,138 |
| 43 | Kremen-1 | KREMEN1 | NM_032045 | 164 | 1,202 | 7.32 | 1,038 |
| 44 | CD40 Ligand | CD40LG | NM_000074 | 533 | 1,562 | 2.93 | 1,028 |
| 45 | CNTF | CNTF | NM_000614 | 911 | 1,928 | 2.12 | 1,017 |
| 46 | IL-19 | IL19 | NM_013371 | 108 | 1,115 | 10.37 | 1,008 |
| 47 | GDF3 | GDF3 | NM_020634 | 633 | 1,616 | 2.56 | 984 |
| 48 | S100 A8/A9 | S100A9 | NM_002965 | 486 | 1,445 | 2.98 | 960 |
| 49 | CNTF R alpha | CNTFR | NM_001842 | 875 | 1,809 | 2.07 | 935 |
| 50 | EG-VEGF / PK1 | PROK1 | NM_032414 | 603 | 1,463 | 2.43 | 860 |
| 51 | FGF-13 1B | FGF13 | NM_004114 | 821 | 1,674 | 2.04 | 853 |
| 52 | CRIM 1 | CRIM1 | NM_016441 | 423 | 1,170 | 2.77 | 747 |
| 53 | Thrombospondin-2 | THBS2 | NM_003247 | 234 | 977 | 4.19 | 744 |
| 54 | MIP 2 | CXCL2 | NM_002089 | 568 | 1,295 | 2.28 | 727 |
| 55 | Angiopoietin-like Factor | ANGPTL7 | NM_021146 | 391 | 1,053 | 2.69 | 662 |
| 56 | uPA | PLAU | NM_002658 | 147 | 805 | 5.48 | 658 |
| 57 | Frizzled-4 | FZD4 | NM_012193 | 202 | 856 | 4.24 | 654 |
| 58 | IGFBP-2 | IGFBP2 | NM_000597 | 13 | 653 | 50.25 | 640 |
| 59 | Tomoregulin-1 | TMEFF1 | NM_003692 | 370 | 1,001 | 2.71 | 631 |
| 60 | FGF-5 | FGF5 | NM_004464 | 232 | 848 | 3.65 | 616 |
| 61 | RELT / TNFRSF19L | RELT | NM_032871 | 157 | 771 | 4.92 | 614 |
| 62 | VEGF | VEGFA | NM_003376 | 41 | 646 | 15.85 | 605 |
| 63 | Chordin-Like 2 | CHRDL2 | NM_015424 | 409 | 992 | 2.43 | 583 |
| 64 | Smad 4 | SMAD4 | NM_005359 | 273 | 800 | 2.93 | 527 |
| 65 | TGF-beta 2 | TGFB2 | NM_001135599 | 151 | 636 | 4.22 | 485 |
| 66 | IL-17 | IL17A | NM_002190 | 400 | 873 | 2.18 | 472 |
| 67 | Activin C | INHBC | NM_005538 | 321 | 790 | 2.47 | 470 |
| 68 | sgp130 | IL6ST | NM_002184 | 89 | 550 | 6.19 | 461 |
| 69 | FGF-11 | FGF11 | NM_006091 | 396 | 852 | 2.15 | 457 |
| 70 | Growth Hormone | GH1 | NM_000515 | 343 | 783 | 2.28 | 440 |
| 71 | EDA-A2 | EDA2R | NM_021783 | 105 | 537 | 5.13 | 432 |
| 72 | TGF-beta 3 | TGFB3 | NM_003239 | 4 | 434 | 123.96 | 430 |
| 73 | MMP-8 | MMP8 | NM_002424 | 152 | 582 | 3.83 | 430 |
| 74 | Siglec-5/CD170 | SIGLEC5 | NM_003830 | 148 | 573 | 3.87 | 425 |
| 75 | PARC / CCL18 | CCL18 | NM_022046 | 267 | 688 | 2.58 | 421 |
| 76 | TNF RII / TNFRSF1B | TNFRSF1B | NM_001066 | 151 | 561 | 3.72 | 410 |
| 77 | NRG2 | NRG2 | NM_004883 | 331 | 741 | 2.24 | 410 |
| 78 | GFR alpha-3 | GFRA3 | NM_001348 | 321 | 726 | 2.26 | 405 |
| 79 | Thrombopoietin | THPO | NM_000460 | 185 | 588 | 3.19 | 403 |
| 80 | Hepassocin | FGL1 | NM_004255 | 375 | 777 | 2.07 | 402 |
| 81 | ROBO4 | ROBO4 | NM_019055 | 110 | 509 | 4.64 | 399 |
| 82 | ErbB3 | ERBB3 | NM_001982 | 55 | 453 | 8.24 | 398 |
| 83 | MCP-2 | CCL8 | NM_005623 | 104 | 498 | 4.79 | 394 |
| 84 | TWEAK / TNFSF12 | TNFSF12 | NM_003809 | 116 | 497 | 4.29 | 381 |
| 85 | PDGF-AA | PDGFA | NM_000860 | 50 | 412 | 8.27 | 362 |
| 86 | Siglec-9 | SIGLEC9 | NM_014441 | 157 | 513 | 3.28 | 357 |
| 87 | Follistatin | FST | NM_005619 | 228 | 583 | 2.56 | 356 |
| 88 | CXCR4 (fusin) | CXCR4 | NM_001008540 | 106 | 451 | 4.25 | 345 |
| 89 | MMP-11 | MMP11 | NM_005940 | 202 | 541 | 2.68 | 339 |
| 90 | FGF-10 / KGF-2 | FGF10 | NM_004465 | 248 | 569 | 2.30 | 321 |
| 91 | Insulysin / IDE | IDE | NM_004969 | 51 | 371 | 7.34 | 320 |
| 92 | GASP-2 / WFIKKN | WFIKKN1 | NM_053284 | 285 | 579 | 2.03 | 294 |
| 93 | BMP-15 | BMP15 | NM_000306 | 219 | 501 | 2.29 | 282 |
| 94 | APJ | AGTRL1 | NM_005161 | 88 | 358 | 4.05 | 270 |
| 95 | TRAIL R2 | TNFRSF10B | NM_003842 | 266 | 535 | 2.01 | 269 |
| 96 | GDF-15 | GDF15 | NM_004864 | 141 | 396 | 2.81 | 255 |
| 97 | TREM-1 | TREM1 | NM_018643 | 152 | 404 | 2.66 | 252 |
| 98 | Nidgen-1 | NID1 | NM_002508 | 63 | 312 | 4.94 | 249 |
| 99 | TRANCE | TNFSF11 | NM_052839 | 109 | 351 | 3.23 | 242 |
| 100 | Kremen-2 | KREMEN2 | NM_024507 | 116 | 353 | 3.05 | 237 |
| 101 | EGF | EGF | NM_000738 | 119 | 353 | 2.97 | 234 |
| 102 | LIF R alpha | LIFR | NM_002310 | 222 | 445 | 2.01 | 224 |
| 103 | Prolactin | PRL | NM_000948 | 156 | 372 | 2.39 | 216 |
| 104 | ICAM-5 | ICAM5 | NM_003259 | 153 | 368 | 2.42 | 216 |
| 105 | MMP-10 | MMP10 | NM_000168 | 104 | 303 | 2.92 | 199 |
| 106 | Smad 7 | SMAD7 | NM_005904 | 144 | 327 | 2.28 | 183 |
| 107 | IL-13 R alpha 1 | IL13RA1 | NM_001997 | 168 | 338 | 2.02 | 171 |
| 108 | Dtk | TYRO3 | NM_006293 | 161 | 329 | 2.04 | 168 |
| 109 | MMP-14 | MMP14 | NM_006708 | 157 | 316 | 2.01 | 159 |
