# Supplementary material for: Paracrine effects of human amniotic epithelial cells protect against chemotherapy-induced ovarian damage
Source: Stem Cell Res Ther. 2017 Nov 28;8:270. doi: 10.1186/s13287-017-0721-0 (PMC5704397; doi:10.1186/s13287-017-0721-0)
Supplement: Supplementary file 4 — This list showed the enriched cytokines in hAECs conditioned medium. These cytokines participate in the regulation of apoptosis (37 cytokines), immune response (34 cytokines), angiogenesis (24 cytokines), or cell cycle progression (16 cytokines). (DOCX 20 kb) [file 13287_2017_721_MOESM4_ESM.docx]

**Additional file 4: Table S3**. This list showed the enriched cytokines in hAECs conditioned medium. These cytokines participate in the regulation of apoptosis (37 cytokines), immune response (34 cytokines), angiogenesis (24 cytokines), or cell cycle progression (16 cytokines).

| **Regulation of apoptosis**  **(37 proteins)** | | **Immune response**  **(34 proteins)** | | **Angiogenesis**  **(24 proteins)** | | **Regulation of cell cycle**  **(16 proteins)** | |
| --- | --- | --- | --- | --- | --- | --- | --- |
| **name** | **Accession**  **Number** | **name** | **Accession**  **Number** | **name** | **Accession**  **Number** | **name** | **Accession Number** |
| **IL6** | NM_000600 | **IL6** | NM_000600 | **IL6** | NM_000600 | **IL6** | NM_000600 |
| **TGFB1** | NM_000660 | **TGFB1** | NM_000660 | **TGFB1** | NM_000660 | **TGFB1** | NM_000660 |
| **TGFB2** | NM_001135599 | **TGFB2** | NM_001135599 | **TGFB2** | NM_001135599 | **TGFB2** | NM_001135599 |
| **VEGFA** | NM_003376 | **VEGFA** | NM_003376 | **VEGFA** | NM_003376 | **VEGFA** | NM_003376 |
| **IL8** | NM_021727 | **IL8** | NM_021727 | **IL8** | NM_021727 | **IL8** | NM_021727 |
| **CD40LG** | NM_000074 | **CSF2** | NM_000758 | **CD40LG** | NM_000074 | **PDGFA** | NM_000860 |
| **CSF3** | NM_001742 | **SMAD4** | NM_005359 | **CSF3** | NM_001742 | **PROK1** | NM_032414 |
| **IL15** | NM_001996 | **SMAD7** | NM_005904 | **IL15** | NM_001996 | **CSF2** | NM_000758 |
| **CCL2** | NM_002982 | **TNFSF11** | NM_052839 | **CCL2** | NM_002982 | **SMAD4** | NM_005359 |
| **MIF** | NM_002415 | **CD40LG** | NM_000074 | **MIF** | NM_002415 | **SMAD7** | NM_005904 |
| **CSF2** | NM_000758 | **CSF3** | NM_001742 | **CXCL1** | NM_005222 | **TNFSF11** | NM_052839 |
| **SMAD4** | NM_005359 | **IL15** | NM_001996 | **PLAU** | NM_002658 | **EGF** | NM_000738 |
| **SMAD7** | NM_005904 | **CCL2** | NM_002982 | **GDF5** | NM_000557 | **S100A9** | NM_002965 |
| **TNFSF11** | NM_052839 | **MIF** | NM_002415 | **MET** | NM_000245 | **TGFB3** | NM_003239 |
| **IL6ST** | NM_002184 | **IL6ST** | NM_002184 | **THBS1** | NM_005930 | **FGF10** | NM_004465 |
| **IFNG** | NM_000619 | **IFNG** | NM_000619 | **TNFSF12** | NM_003809 | **FGF5** | NM_004464 |
| **IGFBP3** | NM_000598 | **IGFBP3** | NM_000598 | **PDGFA** | NM_000860 |  | |
| **IL10** | NM_000572 | **IL10** | NM_000572 | **PROK1** | NM_032414 |  |  |
| **IL17A** | NM_002190 | **IL17A** | NM_002190 | **FGF16** | NM_003868 |  |  |
| **IL19** | NM_013371 | **IL19** | NM_013371 | **FZD4** | NM_012193 |  |  |
| **IL2** | NM_000586 | **IL2** | NM_000586 | **NRG2** | NM_004883 |  |  |
| **IL7** | NM_000880 | **IL7** | NM_000880 | **MMP14** | NM_006708 |  |  |
| **TNFRSF1B** | NM_001066 | **TNFRSF1B** | NM_001066 | **ROBO4** | NM_019055 |  |  |
| **LTA** | NM_000595 | **LTA** | NM_000595 | **THBS2** | NM_003247 |  |  |
| **GDF5** | NM_000557 | **TGFB3** | NM_003239 |  | |  |  |
| **MET** | NM_000245 | **CXCL1** | NM_005222 |  |  |  |  |
| **THBS1** | NM_005930 | **PLAU** | NM_002658 |  |  |  |  |
| **TNFSF12** | NM_003809 | **FST** | NM_005619 |  |  |  |  |
| **EGF** | NM_000738 | **IL13** | NM_002188 |  |  |  |  |
| **S100A9** | NM_002965 | **IL13RA1** | NM_001997 |  |  |  |  |
| **EDA2R** | NM_021783 | **IL21** | NM_021803 |  |  |  |  |
| **GDF15** | NM_004864 | **CCL8** | NM_005623 |  |  |  |  |
| **GZMA** | NM_006144 | **CXCL2** | NM_002089 |  |  |  |  |
| **GH1** | NM_000515 | **CCL18** | NM_022046 |  |  |  |  |
| **IGFBP7** | NM_001553 |  | |  |  |  |  |
| **PRL** | NM_000948 |  |  |  |  |  |  |
| **TNFRSF10B** | NM_003842 |  |  |  |  |  |  |
